# MicroRNAs isolated from peripheral blood in the first trimester predict spontaneous preterm birth

**DOI:** 10.1371/journal.pone.0236805

**Published:** 2020-08-13

**Authors:** Edward E. Winger, Jane L. Reed, Xuhuai Ji, Nardhy Gomez-Lopez, Percy Pacora, Roberto Romero

**Affiliations:** 1 San Francisco, CA, United States of America; 2 Klamath Falls, OR, United States of America; 3 Human Immune Monitoring Center, Stanford University, Stanford, CA, United States of America; 4 Perinatology Research Branch, Division of Obstetrics and Maternal-Fetal Medicine, Division of Intramural Research, Eunice Kennedy Shriver National Institute of Child Health and Human Development, National Institutes of Health, U.S. Department of Health and Human Services, Bethesda, MD, United States of America; 5 Department of Obstetrics and Gynecology, Wayne State University School of Medicine, Detroit, MI, United States of America; 6 Department of Biochemistry, Microbiology and Immunology, Wayne State University School of Medicine, Detroit, MI, United States of America; 7 Department of Obstetrics and Gynecology, University of Michigan, Ann Arbor, MI, United States of America; 8 Department of Epidemiology and Biostatistics, Michigan State University, East Lansing, MI, United States of America; 9 Center for Molecular Medicine and Genetics, Wayne State University, Detroit, MI, United States of America; 10 Detroit Medical Center, Detroit, Michigan, United States of America; 11 Department of Obstetrics & Gynecology, Florida International University, Miami, FL, United States of America; Academic Medical Centre, University of Amsterdam, NETHERLANDS

## Abstract

**Objective:**

To predict spontaneous preterm birth among pregnant women in an African American population using first trimester peripheral blood maternal immune cell microRNA.

**Study design:**

This was a retrospective nested case-control study in pregnant patients enrolled between March 2006 and October 2016. For initial study inclusion, samples were selected that met the following criteria: 1) singleton pregnancy; 2) maternal body mass index (BMI) <30 kg/m^2^; 3) blood sample drawn between 6 weeks to 12 weeks 6 days gestation; 4) live born neonate with no detectable birth defects. Using these entry criteria, 486 samples were selected for study inclusion. After sample quality was confirmed, 139 term deliveries (38–42 weeks) and 18 spontaneous preterm deliveries (<35 weeks) were selected for analysis. Samples were divided into training and validation sets. Real time reverse transcription quantitative polymerase chain reaction (rt-qPCR) was performed on each sample for 45 microRNAs. MicroRNA Risk Scores were calculated on the training set and area-under-the-curve receiver-operating-characteristic (AUC-ROC) curves were derived from the validation set.

**Results:**

The AUC-ROC for the validation set delivering preterm was 0.80 (95% CI: 0.69 to 0.88; p = 0.0001), sensitivity 0.89, specificity of 0.71 and a mean gestational age of 10.0 ±1.8 weeks (range: 6.6–12.9 weeks). When the validation population was divided by gestational age at the time of venipuncture into early first trimester (mean 8.4 ±1.0 weeks; range 6.6–9.7 weeks) and late first trimester (mean 11.5±0.8 weeks; range 10.0–12.9 weeks), the AUC-ROC scores for early and late first trimester were 0.79 (95% CI: 0.63 to 0.91) and 0.81 (95% CI: 0.66 to 0.92), respectively.

**Conclusion:**

Quantification of first trimester peripheral blood MicroRNA identifies risk of spontaneous preterm birth in samples obtained early and late first trimester of pregnancy in an African American population.

## Introduction

The World Health Organization (WHO) has defined preterm birth as those occurring before 37 weeks of gestation [1]. Preterm birth is the leading cause of death in children younger than 5 years of age, accounting for approximately 16% of all deaths, and 35% of deaths among newborn babies (2016 figures) [2]. The annual cost of preterm birth in the United States was estimated at $26.2 billion in 2005 and this number is increasing [3, 4]. Late preterm births experienced an increase of 4% from 2014 through 2016 [5].

Clinically-defined risk factors for preterm birth include a prior history of preterm birth, obesity, hypertension, smoking, diabetes, infection and extremes of maternal age (under 17 or over 40 years old). Laboratory interrogation of urine, cervical mucus, vaginal secretions, serum, plasma and amniotic fluid has been used to supplement clinically defined risk factors to improve prediction of preterm birth. However, the majority of preterm births have no clear risk factor. An individual patient data meta-analysis of 4.1 million singleton births in five high-income countries reported that approximately 65% of all preterm births exhibit none of the 21 pre-specified risk factors [6].

Intense effort has been directed to the discovery of biomarkers that can identify subclinical pathological changes before symptoms and signs of a specific obstetrical syndrome appear. Thus, a wide variety of biomarkers, including proteins, peptides, DNA, RNA, lipids, hormones and cytokines in maternal plasma have been explored for the prediction of preterm birth [7–11]. Currently, investigated biomarkers have not provided adequate sensitivity and specificity to permit intervention at a sufficiently early time point to reach an acceptable risk/benefit profile. WHO has urgently called for new research to identify risk factors that allow for prevention of preterm birth rather than focusing mainly on its management [12].

It has been widely assumed that useful biomarkers are those that are shed into maternal plasma. During pregnancy, plasma is enriched with placentally shed material [13–15]. The syncytiotrophoblast responds to malperfusion by releasing ordinarily secreted substances at abnormal concentrations [16]. Recently, research has been directed at identifying and quantifying proteins in plasma using mass spectrometry. One group demonstrated the existence of predictive proteomic biomarkers (insulin-like growth factor-binding protein 4 (IGFBP4) and sex hormone-binding globulin (SHBG), in maternal plasma [17]. Another group has identified statistically significant circulating microparticle-associated protein biomarker candidates and multiplex panels associated with unique biological processes in their expression profiles at 10–12 weeks among women who subsequently deliver spontaneously at <35 weeks of gestation [18]. These investigators also identified an additional group of proteomic markers in maternal plasma enriched in microparticles that, at the median of 12 weeks gestation, provide clinically useful biomarkers for prediction of spontaneous preterm birth < 35 weeks in a separate, multicenter population [19]. However, the identification of reliable preterm birth biomarkers during the early first trimester has not been demonstrated by these techniques. Quantification of plasma biomarkers representing syncytiotrophoblast-shed material cannot be expected to be informative prior to the initiation of maternal blood perfusion through the intervillous space. Significant placental perfusion is initiated only at the end of the first trimester [20, 21]. In the absence of significant maternal blood flow during the first trimester, pregnancies either destined for a healthy outcome or preterm birth both rely on histiotrophic nutrition and, thereby, do not experience differential syncytiotrophoblast stress due to malperfusion.

We have coined the term “the great obstetrical syndromes" to refer to the most common complications of pregnancy. A fraction of each syndrome is characterized by failure of physiologic transformation of the spiral arteries, which would lead to placental under-perfusion once circulation in the intervillous space is established. [fetal growth restriction (FGR), spontaneous abortion, preeclampsia, preterm labor and preterm premature rupture of the membranes (PPROM)] [22–25]. These conditions share a common pathology characterized by inadequate transformation of the spiral arteries resulting in placental malperfusion once maternal blood flow is established [26–30]. The syncytiotrophoblast responds by shedding a variety of constituents that investigators have quantified in the hope of predicting pregnancy outcome. However, all placentas, whether from patients destined to normal or compromised birth are physiologically hypoxic prior to the initiation of maternal blood flow at the end of the first trimester. Differential syncytiotrophoblast stress, thereby, does not occur [31].

Previous studies by Winger and Reed unexpectedly identified differentially expressed microRNAs within peripheral blood cells between patients who experienced late pregnancy disorders and those that experienced healthy term births [32, 33]. The blood samples were originally collected during the first trimester in a study directed to correlation of blood cell microRNA with functional assays of immune function. Of particular interest, this correlation was found in samples drawn very early during pregnancy (7–9 weeks gestational age) at a time preceding the initiation of placental blood flow thereby preceding placental malperfusion [33]. Later work done by this group found that a first trimester maternal blood microRNA signature predicts risk of spontaneous preterm birth in a white/Asian ethnic population [34]. However, that study did not contain significant numbers of African American women, the population with the highest incidence of preterm birth [35]. The current study expands the microRNA interrogated from 30 to 45 microRNAs and confines the study to an African American population.

## Materials and methods

### Sample collection and consent

This was a retrospective nested case-control study that included samples selected from 11,961 pregnant patients enrolled between March 2006 and October 2016 at the Center for Advanced Obstetrical Care and Research of the Perinatology Research Branch, *Eunice Kennedy Shriver National Institute of Child Health and Human Development (NICHD)*, National Institutes of Health, U.S. Department of Health and Human Services at Hutzel Women’s Hospital, affiliated with the Wayne State University (WSU) School of Medicine and the Detroit Medical Center, Detroit, Michigan. Blood samples were drawn from patients between 6 weeks and 12 weeks 6 days gestational age, prepared as buffy coat by visually-guided pipette aspiration and frozen immediately at -80°C without an RNA preservative. Specimens were maintained frozen for up to eleven years. Specimens were shipped from Wayne State University Detroit, Michigan, USA) directly to the Stanford Human Immune Monitoring Center (Stanford, California, USA) on dry ice where they remained blinded as to clinical outcome through testing, identified only by a unique identification number. All patients provided written informed consent, and the use of biological specimens, as well as clinical and ultrasound data for research purposes, were approved by the Wayne State University Human Investigation Committee.

### Clinical definitions

Gestational age was determined by the last menstrual period and confirmed by ultrasound examination or by ultrasound examination alone if the sonographic determination of gestational age was inconsistent with dating by more than 10 days. “Term from uncomplicated pregnancy” was defined as the delivery of a singleton, normal neonate with the following pregnancy criteria: 1) delivery at 38–42 weeks gestation, 2) birth weight > 10^th^ percentile and < 90^th^ percentile for gestational age (appropriate for gestational age neonate) using the standard proposed by Alexander et al. [36]. Preterm birth was defined as a spontaneous labor (uterine contractions with cervical dilatation leading to delivery) with or without premature rupture of the membranes (PROM) and delivery before 37 weeks of gestation.

### Inclusion criteria

For initial study inclusion, samples met the following criteria: 1) singleton delivery of a live born infant; 2) maternal BMI <30 kg/m^2^; 3) blood sample drawn between 6 weeks and 12 weeks 6 days of gestation; 4) neonate without detectable birth defects. To further focus the analysis on preterm birth versus control differences, we included only preterm birth cases < 35 weeks of gestation.

### MicroRNA selection and analysis

#### Real time PCR

Samples were reverse transcribed using real time quantitative polymerase chain reaction (rt-qPCR) according to the protocol used in our previous studies [33, 37]. Agilent Technologies Human microRNA array kit version 2.4 was utilized according to manufacturer’s instructions to perform microarray analysis [38]. Quantification was recorded as the PCR Ct (Cycle threshold). Ct values inversely correspond to the microRNA concentration whereby Ct represents the number of target amplification cycles required to reach a detection threshold.

#### MicroRNA selection

Forty-five microRNAs were selected for quantification (Table 1, rows 1–45) along with three controls (Table 1, rows 46–48). Thirty of these microRNAs were retained from our previous studies (Table 1, rows 1–30) [32–34] with five added microRNA sequences representing alternate dominant RNA arms (opposite 5' or 3' arm) of the original 30 microRNAs (Table 1, rows 31–35). In addition, 10 additional microRNA sequences were selected (Table 1, rows 31–45) based on their ability to identify risk of adverse pregnancy outcome in an African population (unpublished data. See S1 Table for details). All 45 microRNAs were chosen from a pool of 2,550 microRNAs by differential expression by microarray release 21.0, 8x60K, G4872A-07015 (Agilent Technologies, Santa Clara, California, USA) following labeling performed using the miRNA Complete Labeling and Hybridization Kit 5190–0456 (Agilent Technologies, Santa Clara, California, USA).

**Table 1:** Forty-five microRNAs and three controls were added to each of the twenty-one PCR plates used in the study.

Well No.	microRNA
1	miR-1229-5p
2	miR-1244
3	miR-1267
4	miR-132-3p
5	miR-133b
6	miR-1-3p
7	miR-144-3p
8	miR-146a-5p
9	miR-148a-3p
10	miR-155-5p
11	miR-16-5p
12	miR-181a-5p
13	miR-193a-3p
14	miR-196a-5p
15	miR-199a-5p
16	miR-199b-5p
17	miR-210-3p
18	miR-219-5p
19	miR-221-5p
20	miR-223-5p
21	miR-301a-3p
22	miR-30e-3p
23	miR-33a-5p
24	miR-340-5p
25	miR-424-5p
26	miR-513-5p
27	miR-575
28	miR-582-5p
29	miR-671-5p
30	miR-7-5p
31	miR-181a-3p
32	miR-210-5p
33	miR-221-3p
34	mir-223-3p
35	miR-30e-5p
36	miR-1237-3p
37	miR-1238-3p
38	miR-24-1-5p
39	miR-4485-5p
40	miR-551b-3p
41	miR-6737-3p
42	miR-6752-3p
43	miR-6757-3p
44	miR-6819-3p
45	miR-6889-3p
46	RNU44 (Control)
47	RNU48 (Control)
48	U6 snRNA (Control)

### Real time PCR microRNA plates and quality control

The 486- patient study population was analyzed using twenty-one 48x48 PCR plates (Fluidigm, Biomark, San Francisco, CA, USA) [39]. Twenty-two samples were randomly placed on each plate in duplicate with two control wells. Forty-five microRNAs were quantified for each sample with three RNA controls commonly used for intra-PCR plate microRNA normalization, RNU44, RNU48 and U6 snRNA [40] (Table 1). Quantification was recorded as the PCR Cycle threshold (Ct) at signal detection. RNU48 was selected as the optimal intra-plate control choice based on its ability to demonstrate the most consistent signal with the lowest standard deviation of three RNA controls used in the study (see S2 Table). Patient samples requiring more than 1.0 Ct RNU48 adjustment per plate were excluded from the study. Sample duplicates that did not match within 2.0 Ct of each other were also eliminated from the study. Sample duplicate measurements were averaged to derive the final sample microRNA level.

### Batch effect adjustment

To reduce “batch effect” errors between plates, first, all PCR plates that demonstrated both an RNU48 Ct standard deviation greater than 2.0 Ct per plate and/or a mean plate Ct value greater than 1.5 Ct apart from the 21 plate mean were excluded from the study [four plates out of 21 were eliminated due to these exclusion criteria (S2 Table)]. Though RNU48 was an effective intra-plate control, RNU48 did not follow the microRNA trendline across plates, so was therefore, not used for inter-plate correction. MicroRNA-223-5p was selected for inter-plate correction because; (1) it demonstrated the most consistent signal of 45 microRNAs used in the study; (2) it demonstrated the closest linear correlation with the mean combined microRNA trendline in healthy pregnancy samples across plates (S1 and S2 Figs).

## Results

### Population selection

Using the sample entry criteria described in the Methods section, 486 samples were initially selected for retrospective analysis: 264 healthy, full term deliveries (38.0–42.0 weeks), 69 near term deliveries (37.0–37.9 weeks), 67 spontaneous preterm deliveries without preeclampsia (24.0–36.9 weeks), 77 preeclampsia deliveries (5 were spontaneous preterm deliveries) and 9 medically-indicated preterm deliveries. See Table 2 for the initial population characteristics.

**Table 2:** Initial population for sample analysis*.

Outcome categories	Numbers	Subcategories*	#Samples
**Term (38.0–42.0 weeks)**	**264**	Full term healthy (38.0–42.0 weeks) or Controls	264
**Near term (37.0–37.9 weeks)**	**69**	Near term (37.0–37.9 weeks) (6 SGA)	69
**Preterm (24.0–36.7 weeks)**	**67**	Late preterm (36.0–36.9 weeks)	23
** **	** **	Moderate preterm (34.0–35.9 weeks)	12
** **	** **	Very preterm (24.0–33.9 weeks)	25
** **	** **	Extremely preterm (<24 weeks)	7
**Preeclampsia**	**77**	Late onset preeclampsia (≥34.0 weeks) (5 SPTB including 1 SGA, 16 SGA, 1 HELLP)	67
** **	** **	Early onset preeclampsia (<34.0 weeks) (2 SPTB, 5 SGA, 3 HELLP)	10
**Other**	**9**	Induced preterm delivery	6
** **	** **	Medically-indicated preterm delivery	3
**Total**	**486**		486

*SGA: small-for-gestational age neonate; SPTB: Spontaneous preterm birth; HELLP: Hemolysis, elevated liver enzyme, low platelet count syndrome.

### Study population characteristics

To further focus the analysis on preterm birth versus control differences, we included only preterm birth cases < 35 weeks of gestation. After elimination of samples failing to meet these criteria, 299 samples remained for analysis of the original 486. To reduce the number of co-morbidities present in the final analysis, all “non-spontaneous” preterm deliveries (elective C-section), “near term” (37–38 week) or “late preterm” (35–37 week) deliveries and “preeclampsia” deliveries were removed, resulting in 139 control (38–42 week) and 18 spontaneous preterm (<35 week) delivery samples remaining for final study (Table 3).

**Table 3:** Clinical characteristics of patients who had preterm birth vs. controls.

	Control	Preterm birth	p-value
**No. samples**	139	18	
**Age (years)**	23.0±4.3	24.2±5.8	0.29
**Race**	100% (139/139) black	100% (18/18) black	1.00
**Body mass index (BMI, kg/m**^**2**^**)**	23.6±3.3	23.4±4.2	0.82
**Nullipara**	47% (66/139)	33% (6/18)	0.79
**Prior preterm**	6% (8/139)	56% (10/18)	<0.0001
**Prior spontaneous abortion**	48% (67/139)	44% (8/18)	0.81
**Prior livebirth**	53% (73/139)	67% (12/18)	0.32
**Smoking**	15% (21/139)	17% (3/18)	0.74
**Gestational age at Sampling (weeks)**	10.3±1.8	9.7±1.9	0.19
**Gestational age at delivery (weeks)**	39.6±0.9	29.3±4.3	<0.0001
**Neonate Birthweight (g)**	3352±295	1409±618	<0.0001

*Continuous variables are expressed as mean ± standard deviation. Categorical variables are expressed as percentage (n/N).

### Assignment of samples to training and validation sets

**Fig 1:**
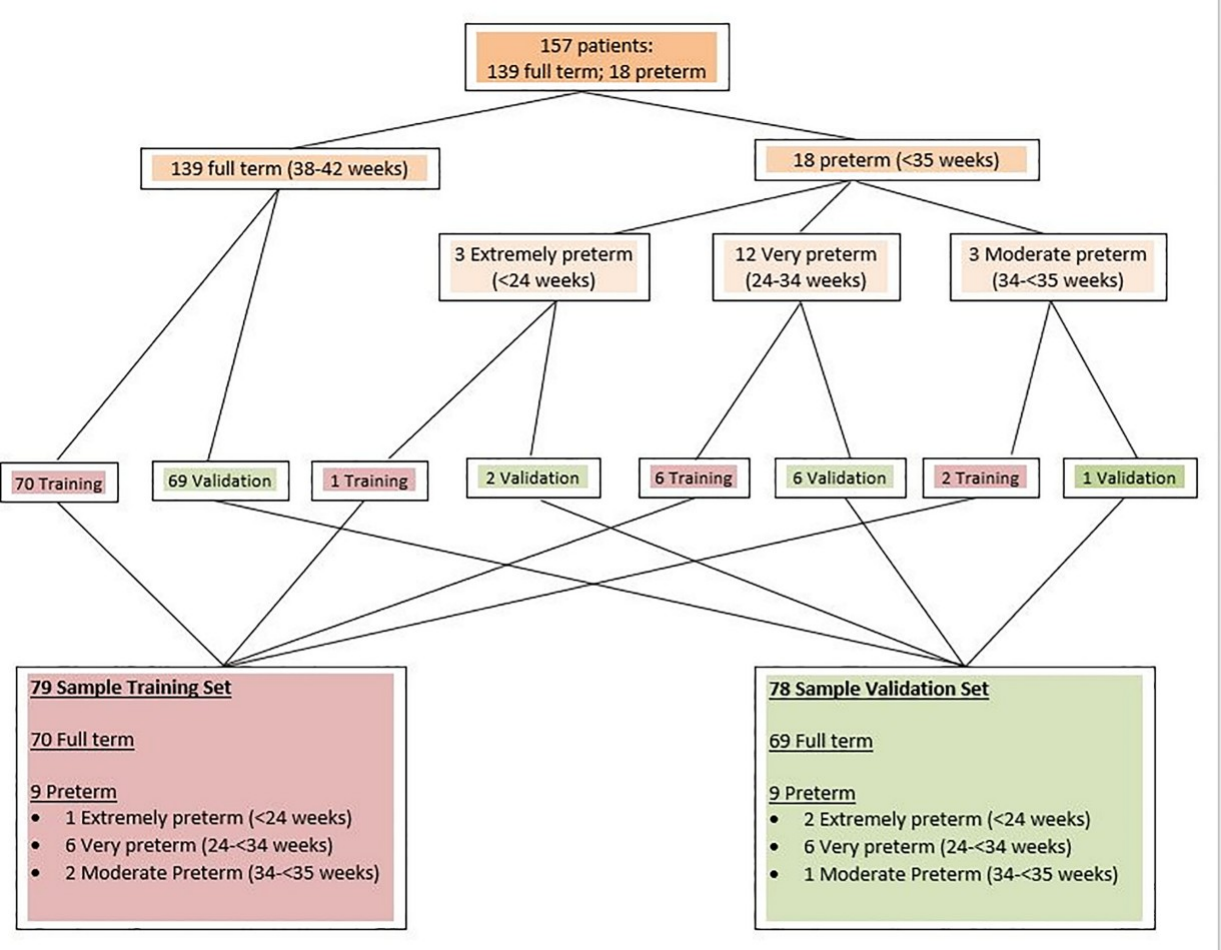
Assignment of samples to training and validation sets. The 157 samples that met the study entry criteria were randomly assigned to training and validation sets. To help assure that the training and validation sets were relatively equivalent, the preterm pregnancies were separately randomized by preterm subgroup: 1) very early preterm (<24 weeks), 2) early preterm (24–34 weeks), and 3) preterm (34–35 weeks). The 79 sample training set was used to develop the microRNA scoring system that was later applied to the 78 sample validation set.

The 157 patient samples that quantified for final analysis were randomized to two groups. The first group designated as the “training set” included 79 first trimester samples from pregnant patients (70 full term 9 preterm). The second group designated as the “validation set” included 78 first trimester patient samples from pregnant patients (69 full term and 9 preterm); (Table 4). To help ensure that the training and validation sets were relatively equivalent, the preterm pregnancies were separately randomized by preterm subgroup: 1) extremely preterm (<24 weeks), 2) very early preterm (24–34 weeks), and 3) moderate preterm (34–35 weeks), (Fig 1). The 79 sample training set was then used to develop the microRNA scoring system that was later applied to the 78 sample validation set.

**Table 4:** Clinical characteristics of the training and validation sets from the preterm birth study group*.

	Training set	Validation set	p value
**No. samples**	79	78	
**Age (years)**	22.9±4.6	23.4±4.3	0.48
**Race**	100% (79/79) black	100% (78/78) black	1.00
**Body mass index (BMI, kg/m**^**2**^**)**	23.0±3.4	24.1±1	0.04
**Nullipous**	51% (40/79)	41% (32/78)	0.26
**Prior preterm**	9% (7/79)	14% (11/78)	0.33
**Prior spontaneous abortion**	41% (32/79)	55% (43/78)	0.08
**Prior livebirth**	49% (39/79)	59% (46/78)	0.26
**Smoking**	19% (15/79)	12% (9/78)	0.27
**Gestational age at sampling (weeks)**	10.5±1.8	10.0±1.8	0.08
**Gestational age at delivery (weeks)**	38.4±3.8	38.4±3.6	1.00
**Baby weight (g)**	3149±667	3131±730	0.87

*Continuous variables are expressed as mean ± standard deviation. Categorical variables are expressed as percentage (n/N).

### Development of the microRNA scoring system using the training set

**Fig 2:**
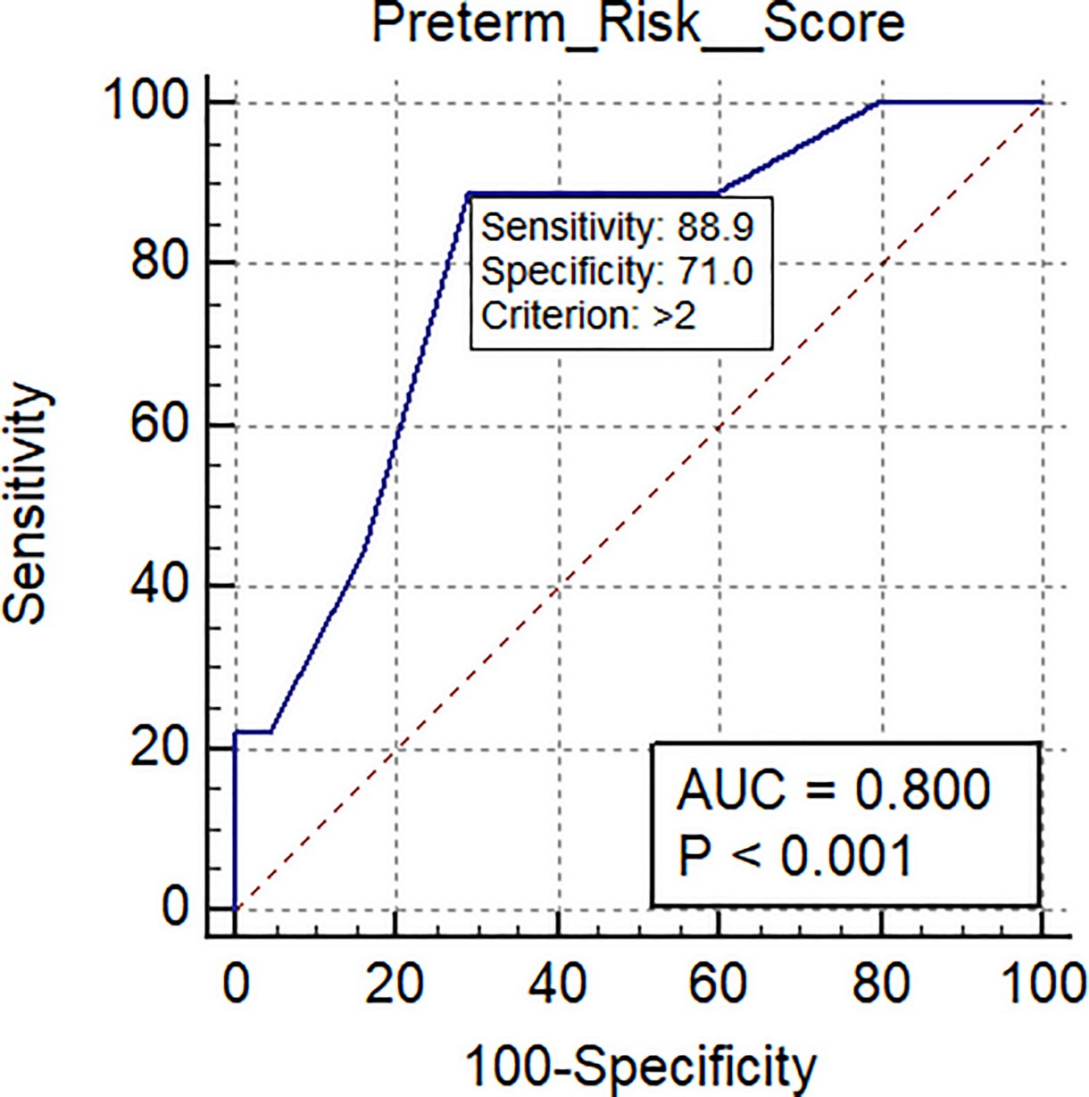
The AUC-ROC score for spontaneous preterm birth for the full first trimester time range (6.6–12.9 weeks gestational age; mean 10.0 ±1.8 weeks) was 0.80 (95% CI: 0.69 to 0.88; p<0.001) in the validation set.

Using the 79-sample training set, an AUC-ROC was calculated for each of the 45 microRNAs using Medcalc® Statistical software (version 19.0.7 Ostend, Belgium) [41]. Only microRNAs where the p value was ≤0.02 were included in the panel: miR-181a-3p, miR-221-3p, miR-33a-5p, miR-6752-3p, miR-1244, miR-148a-3p, miR-1-3p, miR-1267, miR-223-5p, miR-199b-5p, miR-133b and miR-144-3p (Table 5). The Youden Index J Associated Criterion Value for each of the 12 microRNAs was used as its positive/negative cut-off. The panel and calculated cut-off points were then applied to the validation set. Ct values less than the cut-off value for each of the microRNAs in the panel were assigned a score of “1”. The sums of the individual microRNA scores for each patient sample in the validation set were designated as the patient “Risk Score” (Table 6). An AUC-ROC was then calculated from the Risk Scores for the validation set. Using the Risk Scores for the 78 patient samples in the validation set (69 normal delivery; 9 preterm) an AUC-ROC of 0.80 was calculated (95% CI 0.69 to 0.88; p = 0.0001), (Fig 2). The composition of the panel and corresponding cut-offs calculated from the training set were, therefore, deemed validated.

**Table 5:** Selection of the twelve microRNAs for the scoring system using the training set.

#	Selected for Panel	MicroRNA	Sample size	Positive group	Negative group	Area under the ROC curve (AUC)	P value	Associated criterion value	Sensitivity	Specificity
1	x	hsa_miR_181a_3p	71	8	63	0.909	>0.0001	≤12.00	100.00	80.95
2	x	hsa_miR_221_3p	14	3	11	0.909	0.0001	≤28.59	100.00	90.91
3	x	hsa_miR_33a_5p	38	6	32	0.885	0.0001	≤19.72	83.33	90.62
4	x	hsa_miR_6752_3p	39	3	36	0.833	0.0001	>25.02	100.00	72.22
5	x	hsa_miR_1244	68	9	59	0.774	0.0004	≤17.78	66.67	81.36
6	x	hsa_miR_148a_3p	59	7	52	0.808	0.0008	≤14.64	57.14	96.15
7	x	hsa_miR_1_3p	16	3	13	0.846	0.0009	≤23.80	100.00	84.62
8	x	hsa_miR_1267	41	6	35	0.824	0.0009	≤15.92	50.00	100.00
9	x	hsa_miR_223_5p	74	9	65	0.735	0.004	≤15.95	100.00	43.08
10	x	hsa_miR_199b_5p	32	5	27	0.8	0.007	≤24.44	80.00	70.37
11	x	hsa_miR_133b	31	7	24	0.726	0.02	≤26.99	100.00	50.00
12	x	hsa_miR_144_3p	68	8	60	0.733	0.02	≤11.27	50.00	88.33
13		hsa_miR_4485_5p	74	8	66	0.705	0.03	≤12.43	75.00	62.12
14		hsa_miR_340_5p	72	9	63	0.76	0.03	≤15.05	77.78	76.19
15		hsa_miR_132_3p	63	8	55	0.702	0.03	≤15.59	87.50	54.55
16		hsa_miR_219_5p	22	5	17	0.788	0.04	≤24.85	80.00	88.24
17		hsa_miR_424_5p	30	6	24	0.785	0.05	≤20.71	66.67	100.00
18		hsa_miR_221_5p	67	9	58	0.72	0.07	≤14.30	66.67	89.66
19		hsa_miR_1237_3p	51	5	46	0.713	0.07	≤15.37	100.00	43.48
20		hsa_miR_199a_5p	46	7	39	0.755	0.08	≤20.24	71.43	92.31
21		hsa_miR_155_5p	75	9	66	0.677	0.08	≤11.48	88.89	59.09
22		hsa_miR_301a_3p	69	9	60	0.661	0.09	≤12.29	55.56	83.33
23		hsa_miR_575	26	4	22	0.705	0.1	≤25.00	100.00	45.45
24		hsa_miR_6889_3p	59	8	51	0.667	0.1	≤21.21	75.00	64.71
25		hsa_miR_30e_5p	67	9	58	0.669	0.1	≤11.30	44.44	91.38
26		hsa_miR_146a_5p	65	7	58	0.682	0.2	≤10.49	85.71	63.79
27		hsa_miR_30e_3p	67	8	59	0.644	0.2	≤14.09	62.50	62.71
28		hsa_miR_551b_3p	20	5	15	0.707	0.2	≤23.32	40.00	100.00
29		hsa_miR_582_5p	55	8	47	0.67	0.2	≤19.31	50.00	93.62
30		hsa_miR_671_5p	27	3	24	0.681	0.3	≤24.87	100.00	50.00
31		hsa_miR_7_5p	57	8	49	0.648	0.3	≤15.75	50.00	97.96
32		hsa_miR_1229_5p	73	9	64	0.623	0.3	≤9.97	55.56	73.44
33		hsa_miR_6819_3p	14	4	10	0.65	0.4	≤26.49	100.00	40.00
34		hsa_miR_196a_5p	73	9	64	0.573	0.4	≤6.73	77.78	48.44
35		hsa_miR_210_5p	67	9	58	0.571	0.5	≤20.73	77.78	46.55
36		hsa_miR_1238_3p	48	6	42	0.591	0.5	≤22.28	50.00	80.95
37		hsa_miR_6737_3p	69	8	61	0.574	0.6	>21.82	62.50	75.41
38		hsa_miR_193a_3p	62	9	53	0.566	0.6	>23.95	44.44	83.02
39		hsa_mir_223_3p	51	5	46	0.583	0.6	≤4.68	80.00	58.70
40		hsa_miR_181a_5p	52	4	48	0.583	0.7	≤12.03	50.00	93.75
41		hsa_miR_210_3p	61	7	54	0.542	0.7	≤11.07	42.86	81.48
42		hsa_miR_24_1_5p	40	8	32	0.531	0.8	≤25.50	62.50	65.62
43		hsa_miR_6757_3p	39	2	37	0.527	0.9	≤24.49	100.00	32.43
44		hsa_miR_513_5p	0	NA	NA	NA	NA	NA	NA	NA
45		hsa_miR_16_5p	1	NA	NA	NA	NA	NA	NA	NA

“NA” represents microRNA for which an insufficient number of samples were available to generate a ROC curve.

**Table 6:** Spontaneous preterm birth risk assessment using twelve microRNAs to calculate Risk Scores in the validation set.

#	Outcome	Delivery (weeks)	Sample GA (weeks)	miR-181a-3p <12.0	miR-221-3p <28.59	miR-33a-5p <19.72	miR-6752-3p >25.02	miR-1244 <17.77	miR-148a-3p <14.64	miR-1-3p <23.80	miR-1267 <15.92	miR-223-5p <15.95	miR-199b-5p <24.44	miR-133b <27.0	miR-144-3p <11.27	Risk Score
1	Full term	38.9	12.4	13.61		21.05			15.92			15.93			12.66	1
2	Full term	38.9	9.6	12.73		20.46		17.94	16.05			14.99	23.31		11.21	3
3	Full term	40.3	8.1	13.68		20.55		18.49	16.41			16.03	23.28	27.80	12.06	1
4	Full term	40.7	12.4	13.31	29.84	20.90		20.45	15.40			16.28	23.54	29.51	10.60	2
5	Full term	39.6	12.9	14.04	29.91	20.45		19.07	16.32	22.79		16.21	22.72		11.88	2
6	Full term	38.4	8.7	13.98		20.58		21.88	15.88			16.83	24.45		11.98	0
7	Full term	39.3	11.1	11.21		18.91			14.05		19.02	16.06			9.90	4
8	Full term	38	7.1	11.27		18.69		18.25	14.08		18.99	16.09	25.97		11.18	4
9	Full term	39.4	10.7	11.44		17.65		16.41	13.53		18.86	15.59			10.45	6
10	Full term	38.4	9.7			20.91		23.76		26.49		15.79			14.40	1
11	Full term	38.6	12	18.16	29.59	20.78	26.36	22.38			21.64	15.03	28.38		13.19	2
12	Full term	38.3	8.4	21.14		21.64	26.84	23.26	27.17	29.43	23.15	15.77	29.95		14.21	2
13	Full term	41.1	11.7			21.03	26.82	20.54			26.69	15.95		29.52	13.73	2
14	Full term	39.3	11			21.03		20.79			25.82	16.02			15.01	0
15	Full term	38	6.9	12.55		22.67	25.35	18.95	17.95	26.65		15.21	24.17	26.74	12.29	4
16	Full term	38.4	7.7	14.38		22.54	25.18	20.46	17.94	23.46	21.26	15.62	24.85	25.21	11.56	4
17	Full term	40.4	8.4	14.16		22.31		20.36	18.45			15.99	23.62	25.46	12.25	2
18	Full term	38.6	11.1	15.20		23.53	24.76	21.68	18.73		24.53	16.91	25.97	29.10	12.23	0
19	Full term	38	10	13.59		22.46		20.05			20.07	15.60	25.90	27.62	10.50	2
20	Full term	40.3	9.7	14.58		22.92	24.04	20.51	17.77		20.32	15.30	29.49		10.50	2
21	Full term	39.9	9.3					20.16	19.47			15.59	26.46		11.21	2
22	Full term	38.6	9	12.73		22.51	21.86	20.50	17.51		19.20	15.28	27.84		10.59	2
23	Full term	39.6	11.1	13.01		21.87		18.33	19.24	23.25	20.84	14.94	25.60	23.20	14.43	3
24	Full term	40.1	10.9	12.34		21.60		17.71	19.14	19.83	18.94	15.13	25.19	21.51	12.87	4
25	Full term	41.4	9.3	13.13		22.49		18.70	18.73	22.51	18.59	15.59	26.73	23.06	12.48	3
26	Full term	38.9	9.6	12.58		22.01		21.33	16.95	26.97	17.73	15.28	25.19		11.06	2
27	Full term	40.1	8	12.14		22.69	25.52	19.04	18.78		19.26	15.60	26.74	26.56	12.18	3
28	Full term	39.4	12.6	12.26		23.11	24.70	18.25	18.24	21.54	21.71	14.04	23.20	22.16	17.58	4
29	Full term	41	12.7	14.44				17.80	21.06		20.49	15.33		24.51	24.09	2
30	Full term	39.9	9.4	14.49					20.98			17.27			21.15	0
31	Full term	38.3	12.6	13.62				18.90	20.09		20.37	15.80	26.07	24.16	20.56	2
32	Full term	40.6	9.4	14.30			24.49	21.71	20.21		17.76	16.70			20.08	0
33	Full term	38.1	9	12.29			24.20		17.49		15.34	15.27				2
34	Full term	40.1	11.4	13.78		26.16	23.48	18.69	19.51		16.88	15.63			17.53	1
35	Full term	38.1	11.4	11.23			22.08	17.30	17.04		14.37	14.60	24.39		14.53	5
36	Full term	40.7	10.9	14.22			22.75	18.86	19.64		15.47	16.40			14.36	1
37	Full term	39.1	9	14.38				19.19	19.95		17.37	16.16		23.41	18.88	1
38	Full term	41.4	6.7	14.97				18.78	21.29		15.94	16.51		20.22		1
39	Full term	39.3	6.9	11.98				19.14			20.29	15.73			18.52	2
40	Full term	38.3	10	11.10				17.93				14.97			21.02	2
41	Full term	39.6	6.6	12.37			22.96	20.44				14.87			16.33	1
42	Full term	39.7	11.3	13.18			23.04	20.30				16.77			19.38	0
43	Full term	38.1	11	14.44			20.66	20.35	21.36		17.34	16.52			18.77	0
44	Full term	40.3	10.4	14.96			19.99	19.22	22.29		18.86	17.17			22.88	0
45	Full term	40	11	12.20			22.27	16.66	21.84		18.27	14.58	23.48		24.69	3
46	Full term	40.7	11.9	14.24				18.80	22.47		21.17	15.91				1
47	Full term	40.9	8.7	11.15				17.48	18.84			15.48			17.84	3
48	Full term	40	10.9	13.13			21.58					16.70				0
49	Full term	40.7	8.9	11.18				17.20	20.52			14.76			18.60	3
50	Full term	40.1	7	14.14				19.99	22.91			17.11				0
51	Full term	40.7	7.4	11.53			20.09	15.92	20.85		16.46	16.38			16.70	2
52	Full term	40.1	10.4	11.64			18.01	16.47	16.16		16.08	14.76			12.40	3
53	Full term	40.4	11.1				18.31	15.56	18.92			15.07				2
54	Full term	40.4	9.7					16.14								1
55	Full term	40.9	12.6	12.06				16.30			17.49	16.36				1
56	Full term	39.3	11.3	12.45			20.84	18.18	20.29			15.50			17.93	1
57	Full term	40.4	8.1						21.29			15.55			18.14	1
58	Full term	39.3	8.6	11.75			20.94	18.53	19.93		17.21	15.90			13.57	2
59	Full term	38.4	6.9	12.02			21.79	18.15	20.52		18.62	16.95			18.36	0
60	Full term	38.3	9.3	10.10			19.64		15.58		14.94	14.33	23.93	20.86	10.39	6
61	Full term	38.3	12	10.26			19.03	17.11	15.91		15.62	14.18				4
62	Full term	40.4	9	13.24				18.29	21.32			17.03				0
63	Full term	40.4	11.1					18.68	20.81			15.76		18.82	19.34	2
64	Full term	41.3	11.4					19.09	22.11			15.91		20.92	21.97	2
65	Full term	38.9	12.9				23.91	17.92				15.53			18.10	1
66	Full term	40	12.3	13.59			22.02	17.18	19.88			15.22		20.66	20.41	3
67	Full term	40.6	7.9	14.47			21.99	20.98	20.20		19.15	16.78			17.13	0
68	Full term	38.7	12.3													0
69	Full term	40.1	12.1	11.31				17.25	18.68			14.65		21.96	15.44	4
70	Preterm	28.6	7.4	13.97	29.01	20.42		17.63	16.98	23.42		16.78	22.56	24.50	13.02	4
71	Preterm	30.9	11.6	11.61	27.27	19.28	26.57	17.61	14.82	26.66	17.33	15.97	22.51	27.24	10.88	7
72	Preterm	33.1	9.7	12.65		19.67		16.32	15.62		18.63	16.80	23.46		11.58	3
73	Preterm	23.6	7.9	10.48	28.85	19.41	27.96	16.37	14.69	24.79	17.91	15.30	22.64	23.53	10.11	8
74	Preterm	33.3	12.3	14.56		23.96	25.42	22.28	16.62	24.10	21.47	15.82			10.93	3
75	Preterm	34.6	8.4	14.12		22.81	25.33	19.98	18.46	23.41	18.24	15.47	24.89	22.99	12.14	4
76	Preterm	32.4	10.1	12.81			25.52	19.33	20.02	29.60	21.56	15.56	24.48	23.30	24.22	3
77	Preterm	25.9	11.4	13.61			17.58	16.47	18.34			15.15		22.67		3
78	Preterm	23.7	7.3	12.11			23.41	19.21	19.97		20.68	15.51			17.29	1

MicroRNA PCR Ct cut-off values for twelve microRNAs were applied to each patient (designated in rows). A point was given for each microRNA level where the result exceeded the threshold value (shaded cells). The “Risk Score” is displayed in the right-most column.

### Gestational age at time of venipuncture

**Fig 3:**
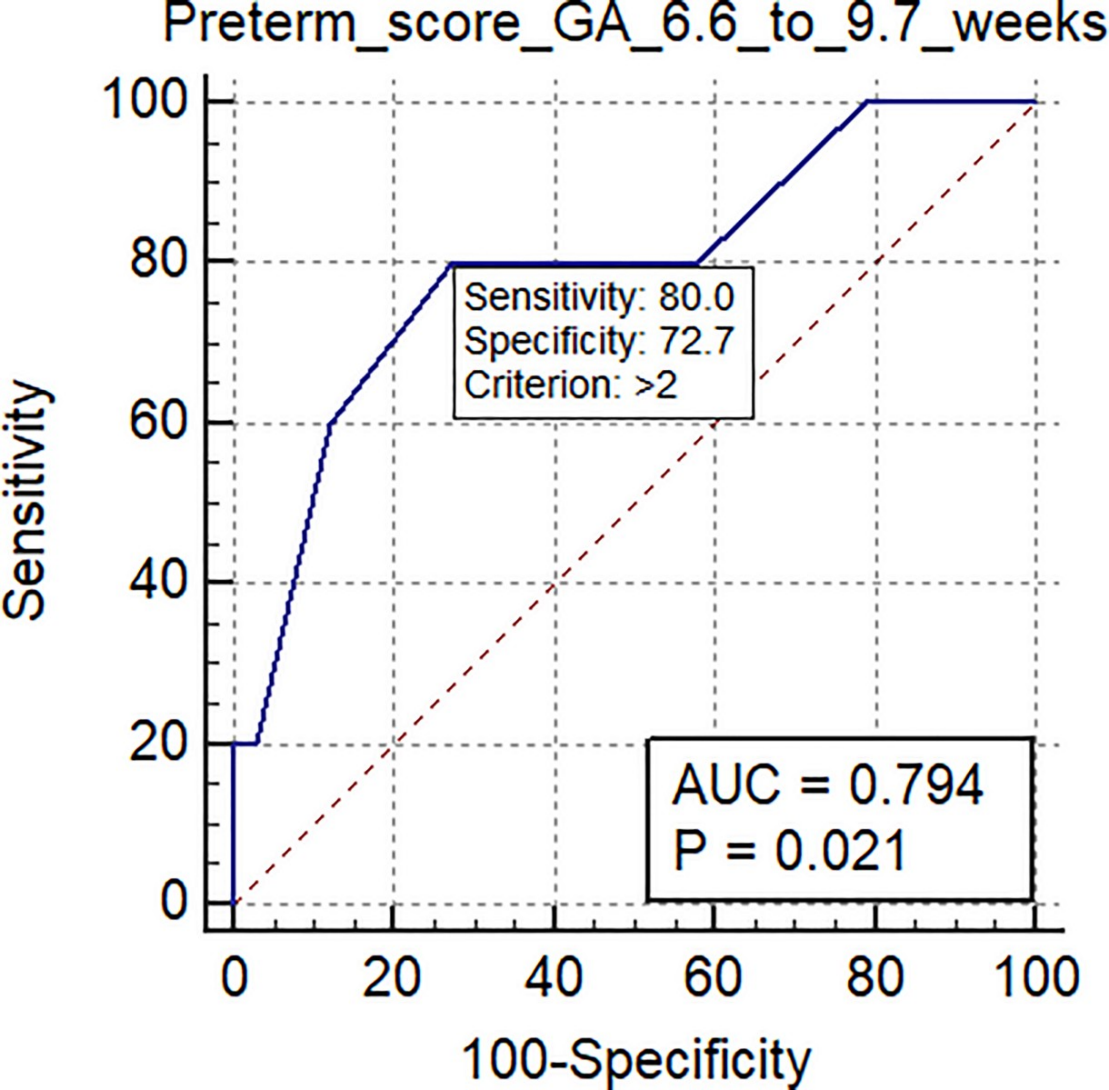
The AUC-ROC score for spontaneous preterm birth in the early first trimester time range (6.6–9.7 weeks gestational age; mean 8.4 ±1.0 weeks) was 0.79 (95% CI: 0.63 to 0.91; p = 0.02) in the validation set.

**Fig 4:**
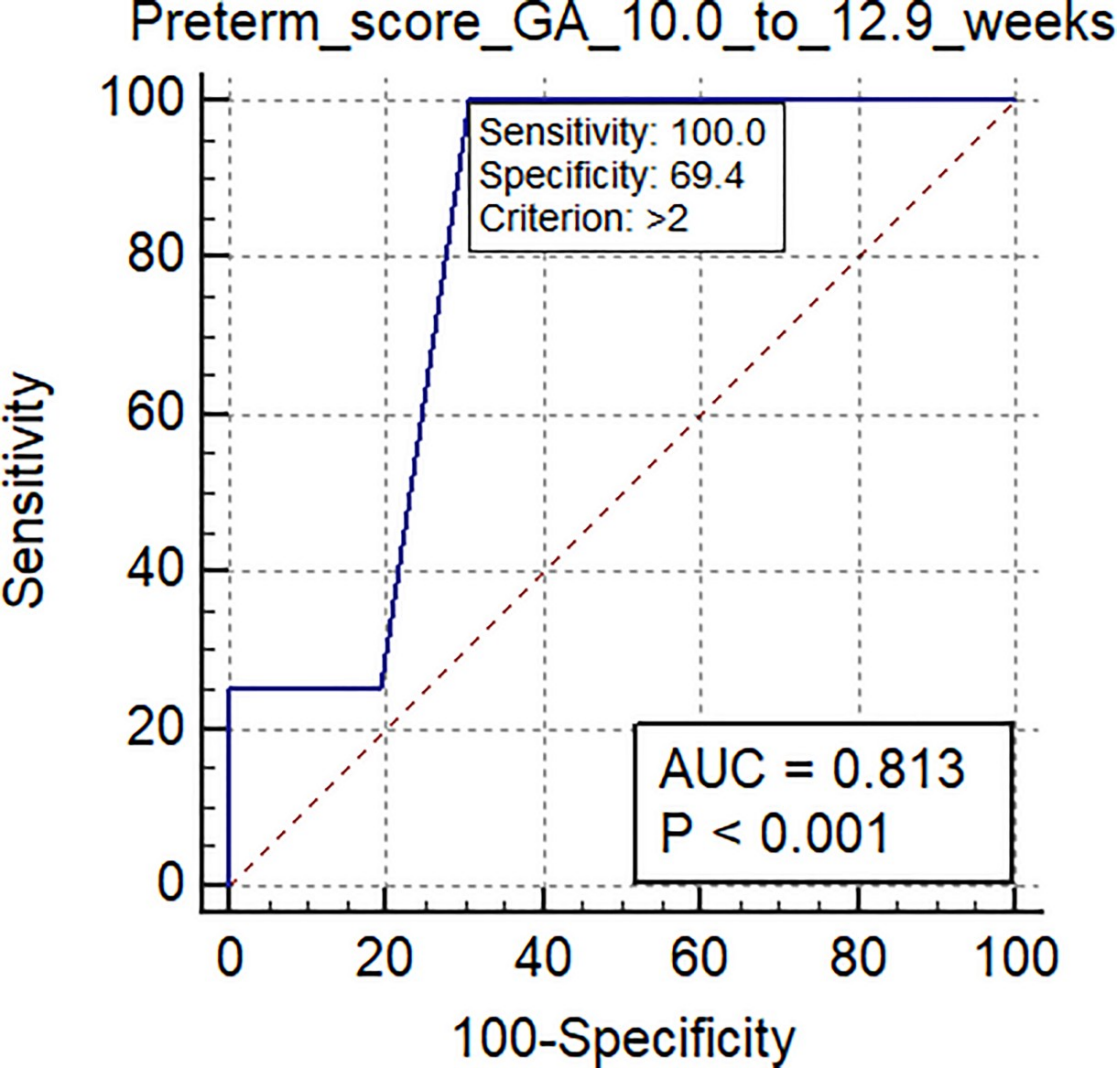
The AUC-ROC score for spontaneous preterm birth in the late first trimester time range (10.0–12.9 weeks gestational age; mean 11.5±0.8 weeks) was 0.81 (95% CI: 0.66 to 0.92; p<0.001) in the validation set.

Mean gestational age of samples in the validation set was 10.0 ± 1.8 weeks gestation (range: 6.6–12.9 weeks). When the validation population was divided into early first trimester (mean 8.4 ±1.0 weeks, range: 6.6–9.7 weeks) and late first trimester (mean 11.5±0.8 weeks, range: 10.0–12.9 weeks) groups, the AUC-ROC scores for the early and late groups were 0.79 (95% CI: 0.63 to 0.91) and 0.81 (95% CI: 0.66 to 0.92), respectively. (Table 7; Figs 3 and 4).

**Table 7:** Scores based on Area Under the surve (AUC)-receiving-operating characteristics curve (ROC) analysis scores for spontaneous preterm birth at different gestational age ranges of sample collection*.

Gest. age range at sample collection	Total 6.6–12.9 weeks (Fig 2)	Early first trimester 6.6–9.7 weeks (Fig 3)	Later first trimester 9.7–12.9 weeks (Fig 4)
Sample size	78	38	40
Positive group (Preterm birth)	9	5	4
Negative group (Healthy)	69	33	36
Area under the ROC curve (AUC)	0.80	0.79	0.81
Standard Error	0.075	0.13	0.081
95% Confidence interval	0.69 to 0.88	0.63 to 0.91	0.66 to 0.92
Significance level P (Area = 0.5)	0.0001	0.0214	0.0001
Sensitivity	89	80	100
Specificity	71	73	69
Positive Predictive Value (PPV)	0.23	0.22	0.24
Negative Predictive Value (NPV)	0.99	0.97	1.00
Positive Likelihood Ratio	3.07	2.96	3.23
Negative Likelihood Ratio	0.15	0.27	0

*Calculations were derived using MedCalc® Statistical Software version 19.0.7 (Ostend, Belgium), except the Likelihood Ratios that were calculated using online software located at: http://getthediagnosis.org/calculator.htm (last accessed 6/13/20) and the PPV, NPV numbers that were calculated using online software located at: http://vassarstats.net/clin2.html (last accessed 6/21/20). Population disease incidence rates used for the PPV and NPV calculations were based on the following reference: Xu X et al. 2009;20(3):729‐747. doi:10.1353/hpu.0.0180.

### Pathways that 12 microRNAs putatively target

Twelve microRNAs identify preterm birth risk in the first trimester of pregnancy with statistical significance. These microRNAs included miR-181a-3p, 221-3p, 33a-5p, 6752-3p, 1244, 148a-3p, 1-3p, 1267, 223-5p, 199b-5p, 133b and miR-144-3p (p≤0.02 for each, using Medcalc® statistical software [40], (Table 5). The top 1,000 genes regulated by these 12 microRNA were selected using mirDip® online analysis software (Jurisica Lab, Krembil Research Institute, Toronto, Canada) [42, 43]. These 1,000 genes then entered into the Reactome® database Version 71 [44] to determine the 20 most statistically significant biological pathways listed in Table 8.

**Table 8:** Twelve microRNA gene pathways analysis*.

No.	Pathway name	# different gene IDs	# total genes	Ratio # total genes/#species genes	P-value	P value adjusted
1.	Membrane Trafficking	94	665	0.046	3.53E-06	6.03E-03
2.	Nuclear Receptor transcription pathway	21	86	0.006	3.19E-05	1.87E-02
3.	FOXO-mediated transcription of cell cycle genes	11	27	0.002	3.28E-05	1.87E-02
4.	Transcriptional Regulation by MECP2	22	100	0.007	9.41E-05	4.02E-02
5.	FOXO-mediated transcription	23	110	0.008	1.37E-04	4.70E-02
6.	Ion channel transport	35	207	0.014	1.99E-04	4.93E-02
7.	Regulation of MECP2 expression and activity	12	39	0.003	2.02E-04	4.93E-02
8.	Circadian Clock	21	104	0.007	4.11E-04	6.89E-02
9.	SUMO E3 ligases SUMOylate target proteins	31	183	0.013	4.33E-04	6.89E-02
10.	SUMOylation	32	192	0.013	4.63E-04	6.89E-02
11.	RAF activation	11	37	0.003	4.85E-04	6.89E-02
12.	Gene expression (Transcription)	200	1,850	0.128	5.74E-04	7.27E-02
13.	Estrogen-dependent gene expression	27	154	0.011	5.96E-04	7.27E-02
14.	RUNX1 regulates expression of components of tight junctions	5	8	0.001	7.41E-04	8.45E-02
15.	RNA Polymerase II Transcription	183	1,692	0.117	9.92E-04	1.03E-01
16.	ESR-mediated signaling	38	256	0.018	1.22E-03	1.03E-01
17.	Cyclin D associated events in G1	12	48	0.003	1.22E-03	1.03E-01
18.	G1 Phase	12	48	0.003	1.22E-03	1.03E-01
19.	RUNX1 regulates transcription of genes involved in WNT signaling	5	9	0.001	1.24E-03	1.03E-01
20.	Activation of BH3-only proteins	10	36	0.002	1.42E-03	1.03E-01

*Pathways and statistical calculations derived using Reactome® online database and software found at: https://reactome.org/ (last accessed 1/20/20; ref: Jassal B et al. The reactome pathway knowledgebase. Nucleic Acids Res. 2019 Nov 6. doi: 10.1093/nar/gkz1031. PubMed PMID: 31691815).

## Discussion

### Principal findings of the study

Our results indicate that first trimester maternal blood microRNA identifies the risk of spontaneous preterm birth in blood samples drawn at both early and late first trimester time points in an African American population. The AUC-ROC for preterm birth (<35 weeks) was 0.80 (95% CI: 0.69 to 0.88; p = 0.0001) in specimens collected at a mean of 10.1 ±1.8 weeks gestation (range: 6.6–12.9 weeks).

### Results in the context of what is known

The results of the current research are consistent with previous studies published by our group that have demonstrated that peripheral blood cell microRNA obtained during the first trimester of pregnancy predicts risk of preterm delivery [34], spontaneous abortion [37, 33], and preeclampsia [32]. In addition, the current study confirms the usefulness of peripheral blood cell microRNA as biomarkers identifying risk of preterm birth early in the first trimester. When the study validation set was divided into early first trimester and late first trimester groups, the AUC-ROC scores for the early and late first trimester groups were 0.79 (95% CI: 0.63 to 0.91) and 0.81 (95% CI: 0.66 to 0.92), respectively. No other biomarkers are predictive so early within the first trimester to date.

### Clinical implications

A clinically useful tool for predicting the risk of preterm birth should demonstrate adequate sensitivity and specificity at a time point when successful intervention can be initiated. Low dose aspirin and/or antiplatelet therapies (dipyridamole, heparin) have demonstrated efficacy at reducing preterm birth rates and related pregnancy complications, but only when started early in pregnancy [45, 46]. Low dose aspirin therapy (80mg daily) started at 8–16 weeks gestation has been found to result in a 35% reduction in preterm birth while losing its efficacy when instituted later [47]. Sweeting et al. note that blood testing performed during the same time window as non-invasive prenatal testing utilizing plasma DNA (NIPT) could provide management guidance from the outset of pregnancy [48]. MicroRNA testing could be included as a part of an “inverted pyramid” of pregnancy care where more active assessment of risk is performed early in pregnancy as suggested by other authors [49]. No other biomarkers are predictive so early within the first trimester to date.

Preterm birth rates in the USA are highest in African American infants [35]. The reasons for this disparity are poorly understood. It has been suggested that differences in preterm birth rates cannot be explained solely by sociodemographic factors [50]. The added role of genetics/epigenetics in preterm birth in African American women is supported by persuasive epidemiologic data [51, 52]. In addition, the high predictive power of immune cell microRNA quantification reported by Winger and Reed support genetic/epigenetic components in the pathogenesis of preterm birth in a white and Asian population [34].

In addition to genetic/epigenetic factors, other covariates may be associated with the predictive value of the 12-microRNA panel. When other covariates (BMI, smoking, miscarriage, etc.) were considered in our current study (Table 3), most were not found to be significantly associated with differential pregnancy outcome, with the exception of prior preterm birth history, which was found to be strong “contributor” to preterm birth prediction (p<0.0001). To investigate the relationship between prior spontaneous preterm birth and the microRNA risk scores, we removed all women in our study that had a history of preterm birth for purposes of inquiry. With the preterm birth covariate removed, the 12- microRNA panel, still identified preterm birth risk with significance, (AUC-ROC 0.76, p<0.01; sensitivity 0.83, specificity of 0.69. (See S3 Fig). This suggests that the 12-microRNA panel may work effectively in women with or without a preterm birth history.

Though our current study focused on mid to early preterm birth prediction (< 35 weeks gestation), preliminary data suggest that microRNA markers can also predict late preterm birth (> 35 weeks). When our nested study population was expanded (for inquiry purposes) to include preterm birth defined at <36 weeks for both the training and validation sets, the AUC-ROC still remained significant at 0.77, (p<0.001; see S4 Fig). Winger and Reed previously published data demonstrating microRNA marker effectiveness predicting late preterm birth in a white/Asian population up to 37 weeks 6 days gestation [34]. These combined findings offer hope that maternal cell microRNA markers eventually will be shown effective at predicting both early and late spontaneous preterm birth. Further investigations are needed to confirm this idea.

### Research implications

Spiral arteries within the placental bed are largely plugged by trophoblast prior to 12 weeks of gestation maintaining the placenta and fetus in a relative state of physiologic hypoxia through week 14 of gestation [53]. Only after 14 weeks of pregnancy do the trophoblast plugs fully dissipate initiating blood flow at the beginning of the second trimester [54]. With increasing oxygen concentrations, extravillous trophoblast mature from a proliferative to a non-proliferative, invasive phenotype enhancing spiral artery transformation [55]. Prior to the establishment of blood flow, however, we would not expect to find shed placental biomarkers differentially released into maternal plasma. Quantification of peripheral blood cell microRNA has offered a new approach.

Winger and Reed suggested an alternative to interrogation of plasma components. They hypothesized that interrogation of the maternal immune cell component of the placental bed would offer at least two significant advantages: first, the method would eliminate the interrogation of an intermediary organ, the placenta, rather than direct interrogation of the underlying pathology in the placental bed and, second, the method would allow recognition of the underlying pathology at an earlier time point [32–34, 37]. However, investigation of the placental bed is more challenging than examination of placental shed components. Acquiring placental bed immune cells at an early time-point is prohibitively risky and difficult to justify, particularly in low risk populations.

Winger and Reed proposed using peripheral blood cells as surrogates for immune cells in the placental bed [34]. Historically, interrogation of peripheral blood immune cells as surrogates for placental bed immune cells has not found support [56]. Peripheral blood cells such as natural killer (NK) cells are significantly different in both phenotype and function than corresponding NK cells within the placental bed [57]. Were a large repertoire of biomarkers shared by both circulating and placental-bed resident immune cells, the expression of some fraction of such a repertoire might be regulated in a corresponding manner. Pelosi et al. recently published data demonstrating a high correlation between the microRNA expression levels of peripheral blood NK and decidual NK cells (r = 0.96) [58].

Of the thousands of microRNAs expressed by peripheral blood cells, we hoped to identify a subset that might correlate with immune cell activity within the placental bed. In the current study, we identified 12 microRNAs that could identify spontaneous preterm birth risk (p≤0.02 for each) (Table 5). The top 1,000 genes regulated by these 12 microRNAs were selected using mirDip® online software then entered into the Reactome® database to select 20 most statistically significant biological pathways. These pathways are listed in Table 8. Many of these pathways are involved in the regulation of membrane trafficking, cell growth and cell proliferation. As listed, the four most significant pathways included: (1) “Membrane Trafficking”, (2) “Nuclear Receptor transcription pathway,” and (3) “Forkhead box class O (FOXO)- mediated transcription” and (4) “Transcriptional regulation by methyl-cytosine binding protein-2 (MECP2)”. Interestingly, two of the top 20 pathways found in our analysis were associated with estrogen regulation of cellular proliferation and differentiation (“Estrogen Signaling Receptor (ESR)-mediated signaling” and “Estrogen-dependent gene expression”). These pathways are consistent with the important role that estrogen plays in the implantation process in early pregnancy [59]. Estrogen and progesterone both regulate the recruitment, proliferation, differentiation and function of decidual NK cells in the uterus during early pregnancy [60]. Decidual natural killer cells, macrophages and Innate Lymphoid cells (ILCs) are known to be involved in implantation [61–65]. The progenitors of these cells may migrate to the decidua from the peripheral blood [66, 67]. Of the 12 microRNAs selected for the microRNA panel (Table 5), seven were also found to identify the risk of adverse pregnancy outcome in our previously published pregnancy microRNA panels: miR-181a-3p, -221, -33a-5p, -148a-3p, -1-3p, -1267 and -223-5p [32–34,37]. MiR-181a-3p is known to be dysregulated in the vascular endothelium of placental villi of unhealthy pregnancy, [68] miR-221-3p is dysregulated in peripheral blood of women with preeclampsia [69] and miR-33a is dysregulated women with metabolic syndrome [70, 71]. In addition, miR-148a-3p, was found by Manaster et al. to potentially target the 3’UTR (3 prime untranslated region) of HLA-G, a leukocyte antigen which has a critical role in maternal tolerance of the fetus [72]. These known pregnancy associations support the importance of the microRNA that we have identified. However, future studies are warranted to confirm these microRNA pathways and mechanisms. Expression of differentially expressed microRNA within placental bed immune cells by in situ hybridization also needs to be explored.

### Strengths and limitations

Strengths of this study include the following: 1) all specimens were blinded to the lab performing the microRNA quantification; 2) all specimens were run in duplicate. Only well-matched duplicates were included in the final calculations; 3) all results were confirmed using training and validation sets; 4) a large number of samples were used given the number of microRNAs tested, increasing statistical power; 5) both early and late first trimester time-frame subgroups yielded similar, yet independent, powerful AUC ROCs, and 6) eleven of the twelve microRNAs most able to identify pregnancy outcome signaled in a common direction, indicating the presence of common (non-random) underlying biology.

Limitations of this study include the following: 1) the study was retrospective rather than prospective, 2) frozen samples were used, not fresh; 3) a single patient ethnic group was used (African American) which may limit the generalizability of the results; 4) this was a nested case-control study where disease “prevalence,” is artificially high so the resulting positive/negative and AUC values might not reflect those seen in a natural population; 5) there was no external validation of results; 6) there was a loss of specimens due to (a) elimination of PCR plates that performed poorly and (b) elimination of duplicate specimens that did not match. However, because these performance issues were present in both the preterm and healthy subgroups equally, they should not alter the final conclusions.

## Conclusions

Quantification of first trimester peripheral blood microRNA predicts risk of spontaneous preterm birth in samples drawn in the early and late first trimester in an African American population. Immune cell microRNA quantification may permit the clinician to institute early intervention at a time when therapy is most effective.

## Supporting information

S1 File Supplementary data file containing sample data and microRNA Ct expression levels. (XLSX)

S1 Table Selection of 10 additional microRNAs to include in preterm birth study. (DOCX)

S2 Table Data calculations used for RNA control selection for intra-plate normalization adjustments for individual PCR plates. (DOCX)

S1 Fig Mean rt PCR Ct (cycle threshold) values among microRNAs and controls in healthy pregnancy across 21 plates for inter-plate control selection. (DOCX)

S2 Fig Mean rt qPCR Ct readings with trendlines in healthy pregnancy across 21 plates. (DOCX)

S3 Fig Preterm AUC-ROC for 12-microRNA panel in women with no prior history of preterm birth. (DOCX)

S4 Fig Preterm AUC-ROC in population expanded to include women with late preterm birth (<36 weeks). (DOCX)
