# Supplementary material for: MicroRNAs isolated from peripheral blood in the first trimester predict spontaneous preterm birth
Source: PLoS One. 2020 Aug 13;15(8):e0236805. doi: 10.1371/journal.pone.0236805 (PMC7425910; doi:10.1371/journal.pone.0236805)
Supplement: S1 Table — (DOCX) [file pone.0236805.s002.docx]

**S1 Table.** **Selection of 10 microRNAs** **to predict adverse pregnancy outcome**

| **No.** | **Top 10 microRNAs**  **from 2,550 in microArray***  **(A)** | **Ratio**  **(B)** | **AUC-ROC**  **(C)** | **p value <0.05**  **(D)** | **Mean Signal Strength**  **>5.0 Ct**  **(E)** | **Signal**  **Consistency**  **(>85% samples)**  **(F)** |
| --- | --- | --- | --- | --- | --- | --- |
| 1 | **hsa-miR-4485-5p** | 1.89 | 0.94 | <0.0001 | 301.95 | x |
| 2 | **hsa-miR-551b-3p** | 1.47 | 0.83 | 0.0455 | 131.01 | x |
| 3 | **hsa-miR-24-1-5p** | 1.53 | 0.83 | 0.0455 | 25.23 | x |
| 4 | **hsa-miR-6819-3p** | 1.77 | 0.89 | 0.0031 | 15.58 | x |
| 5 | **hsa-miR-1238-3p** | 2.06 | 0.94 | <0.0001 | 11.69 | x |
| 6 | **hsa-miR-6737-3p** | 2.19 | 0.83 | 0.0455 | 10.76 | x |
| 7 | **hsa-miR-1237-3p** | 1.77 | 0.89 | 0.0031 | 10.36 | x |
| 8 | **hsa-miR-6757-3p** | 1.66 | 0.83 | 0.0455 | 10.26 | x |
| 9 | **hsa-miR-6889-3p** | 2.03 | 0.89 | 0.0017 | 9.43 | x |
| 10 | **hsa-miR-6752-3p** | 1.65 | 0.89 | 0.0031 | 5.23 | x |

* Top 10 microRNAs of 2,550 microRNAs selected by differential expression by microarray release 21.0, 8x60K, G4872A-07015 (Agilent Technologies, Santa Clara, California, USA) following labeling performed using the miRNA Complete Labeling and Hybridization Kit 5190-0456 (Agilent Technologies, Santa Clara, California, USA).

**Selection of 10 microRNAs for adverse pregnancy outcome prediction**

To select these additional 10 microRNAs most predictive of pregnancy outcome, a series of steps were performed:

1. Blood samples taken from nine pregnant women of African ancestry in their first trimester (11-13 weeks of pregnancy) and retrospectively evaluated (three healthy pregnancies and six “compromised” pregnancies” including preeclampsia, preterm and/or fetal growth restriction).
2. 2,550 microRNAs were isolated and quantified according to the procedure given in the paper by Winger at al. (Winger, et al. PLoS One. 2018 Jan 2;13(1):e0190654) using Agilent's GeneSpring GX v11.5.1. kit available from: http://genespring-support.com/resources/documentation (last accessed 11/3/2019).
3. Identification of suitable candidate microRNAs was based on a p value of <0.05 on ROC analysis for compromised pregnancy outcome prediction (MedCalc Statistical Software version 18.10.2 (MedCalc Software bvba, Ostend, Belgium; http://www.medcalc.org; 2018). Because ROC curve analysis for 2,550 microRNA exceeded our capacity for individual ROC analysis, we pre-selected suitable candidate microRNAs where the numerator comprises the difference between the mean Ct value of the “compromised” population minus the mean value of the “healthy” population and the denominator comprises the average of the two standard deviations of the values for healthy and compromised individuals to calculate a Ratio (Column B) that equaled or exceeded 1.3. ROC analyses were then performed (Column C). MicroRNAs with qualifying p values were selected (Column D).
4. MicroRNAs were also selected based on suitability for clinical use. Those with a mean signal strength greater than 5.0 Ct (Column E) and appropriate signal consistency among patient samples (85% of patient samples demonstrate a measurable signal) were selected (Column F)
5. After applying this list of selection criteria, 10 microRNAs were selected as being most clinically promising and were added to the panel. These miRNAs include hsa-miR-4485-5p, hsa-miR-551b-3p, hsa-miR-24-1-5p, hsa-miR-6819-3p, hsa-miR-1238-3p, hsa-miR-6737-3p, hsa-miR-1237-3p, hsa-miR-6757-3p, hsa-miR-6889-3p and hsa-miR-6752-3p (Column A).
