# Supplementary material for: MicroRNAs isolated from peripheral blood in the first trimester predict spontaneous preterm birth
Source: PLoS One. 2020 Aug 13;15(8):e0236805. doi: 10.1371/journal.pone.0236805 (PMC7425910; doi:10.1371/journal.pone.0236805)
Supplement: S2 Table — (DOCX) [file pone.0236805.s003.docx]

**S2 Table.** Data calculations to help determine the normalization adjustment per individual PCR plate.

| **Plate No.** | **RNA Controls** | **Mean Ct /Chip** | **SD** | **X=Excluded plate (Plates with RNU48 SD > 2.0 Ct and/or a mean plate Ct > 1.5 Ct from the 21 plate mean)** |
| --- | --- | --- | --- | --- |
| **Plate 1** | RNU48 | 11.84045 | 4.584993 | x |
|  | RNU44 | 12.84943 | 4.794458 |  |
|  | U6 snRNA | 19.53862 | 2.810147 |  |
| **Plate 2** | RNU48 | 6.410063 | 0.939806 |  |
|  | RNU44 | 9.456269 | 1.564228 |  |
|  | U6 snRNA | 18.57624 | 1.343749 |  |
| **Plate 3** | RNU48 | 7.564532 | 0.884007 |  |
|  | RNU44 | 9.601313 | 0.975062 |  |
|  | U6 snRNA | 17.89669 | 3.363508 |  |
| **Plate 4** | RNU48 | 12.30477 | 2.122775 | x |
|  | RNU44 | 16.68872 | 2.626107 |  |
|  | U6 snRNA | 18.63858 | 3.313573 |  |
| **Plate 5** | RNU48 | 6.780136 | 0.937646 |  |
|  | RNU44 | NA | NA |  |
|  | U6 snRNA | 16.2846 | 1.111449 |  |
| **Plate 6** | RNU48 | 7.101617 | 0.851333 |  |
|  | RNU44 | 13.28902 | 1.157687 |  |
|  | U6 snRNA | 19.20632 | 1.103441 |  |
| **Plate 7** | RNU48 | 10.85766 | 2.587425 | x |
|  | RNU44 | 13.22421 | 2.212623 |  |
|  | U6 snRNA | 20.3657 | 1.875527 |  |
| **Plate 8** | RNU48 | 6.53073 | 1.253576 |  |
|  | RNU44 | 15.9657 | 2.259768 |  |
|  | U6 snRNA | 20.64187 | 1.773028 |  |
| **Plate 9** | RNU48 | 7.554586 | 0.995457 |  |
|  | RNU44 | 13.1364 | 0.660199 |  |
|  | U6 snRNA | 20.3838 | 1.274268 |  |
| **Plate 10** | RNU48 | 7.227454 | 1.102278 |  |
|  | RNU44 | 12.2043 | 1.173548 |  |
|  | U6 snRNA | 19.99111 | 1.255364 |  |
| **Plate 11** | RNU48 | 8.597089 | 1.474109 |  |
|  | RNU44 | 14.10747 | 1.87975 |  |
|  | U6 snRNA | 19.29346 | 1.178834 |  |
| **Plate 12** | RNU48 | 11.30277 | 1.514096 |  |
|  | RNU44 | NA | NA |  |
|  | U6 snRNA | 19.52047 | 1.070513 |  |
| **Plate 13** | RNU48 | 6.073673 | 0.974186 |  |
|  | RNU44 | NA | NA |  |
|  | U6 snRNA | 18.76124 | 0.737917 |  |
| **Plate 14** | RNU48 | NA | NA |  |
|  | RNU44 | NA | NA |  |
|  | U6 snRNA | 21.01813 | 1.340238 |  |
| **Plate 15** | RNU48 | 7.756966 | 1.426985 |  |
|  | RNU44 | 13.79516 | 1.271559 |  |
|  | U6 snRNA | 21.09692 | 1.158876 |  |
| **Plate 16** | RNU48 | 7.130278 | 1.219565 |  |
|  | RNU44 | 18.07024 | 1.890262 |  |
|  | U6 snRNA | 20.16715 | 1.661822 |  |
| **Plate 17** | RNU48 | 7.530391 | 1.050651 |  |
|  | RNU44 | 15.80155 | 1.296999 |  |
|  | U6 snRNA | 21.07353 | 1.356746 |  |
| **Plate 18** | RNU48 | 8.800815 | 1.694241 |  |
|  | RNU44 | NA | NA |  |
|  | U6 snRNA | 21.11159 | 1.829892 |  |
| **Plate 19** | RNU48 | 7.791355 | 1.174657 |  |
|  | RNU44 | NA | NA |  |
|  | U6 snRNA | 22.83264 | 1.941749 |  |
| **Plate 20** | RNU48 | 10.86634 | 2.708898 | x |
|  | RNU44 | 13.62627 | 2.19927 |  |
|  | U6 snRNA | NA | NA |  |
| **Plate 21** | RNU48 | 9.592288 | 1.068143 |  |
|  | RNU44 | 16.55113 | 0.744354 |  |
|  | U6 snRNA | 19.68434 | 0.779606 |  |

** “NA” represents an RNA control for which an insufficient number of samples generated a meaningful signal on the PCR Plate.

Each of the 21 chips contains three RNU controls: RNU48, RNU44 and U6snRNA. As seen in the data table below, RNU48 signaled most consistently (fewest non-signal readings) for the 21 chips so was used as the primary control for intra-plate normalization. PCR plates that demonstrated both an RNU48 Ct standard deviation greater than 2.0 Ct per plate and/or a mean plate Ct value greater than 1.5 Ct apart from the 21-plate combined mean were excluded from the study.
