# Supplementary material for: MicroRNAs isolated from peripheral blood in the first trimester predict spontaneous preterm birth
Source: PLoS One. 2020 Aug 13;15(8):e0236805. doi: 10.1371/journal.pone.0236805 (PMC7425910; doi:10.1371/journal.pone.0236805)
Supplement: S1 Fig — (DOCX) [file pone.0236805.s004.docx]

**S1 Figure.** Mean PCR Ct (cycle threshold) values among microRNAs and controls in healthy pregnancy outcome across 21 Plates.


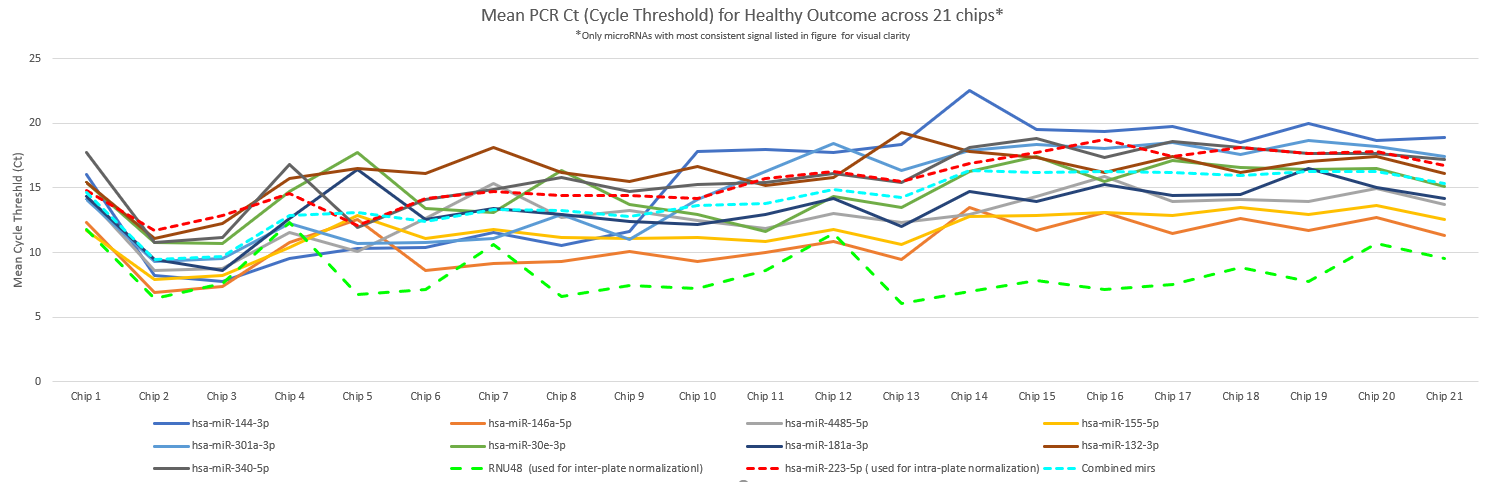


**S1 Figure.** Mean rt qPCR Ct (cycle threshold) readings for top 9 signaling microRNAs of 45 in healthy pregnancy outcome across 21 plates. The dashed line represents miR-223-5p, the light green dashed line represents the RNU48 control and the light blue dashed line represents the combined average of the microRNA readings across plates. As seen here, miR223-5p line followed the microRNA average across the 21 plates and was thus selected for inter-plate control.
