# Supplementary material for: MicroRNAs isolated from peripheral blood in the first trimester predict spontaneous preterm birth
Source: PLoS One. 2020 Aug 13;15(8):e0236805. doi: 10.1371/journal.pone.0236805 (PMC7425910; doi:10.1371/journal.pone.0236805)
Supplement: S2 Fig — (DOCX) [file pone.0236805.s005.docx]

**S2 Figure.** Mean rt qPCR Ct (cycle threshold) readings for healthy pregnancy outcome across 21 plates (dashed lines) with associated trendlines (dotted lines).


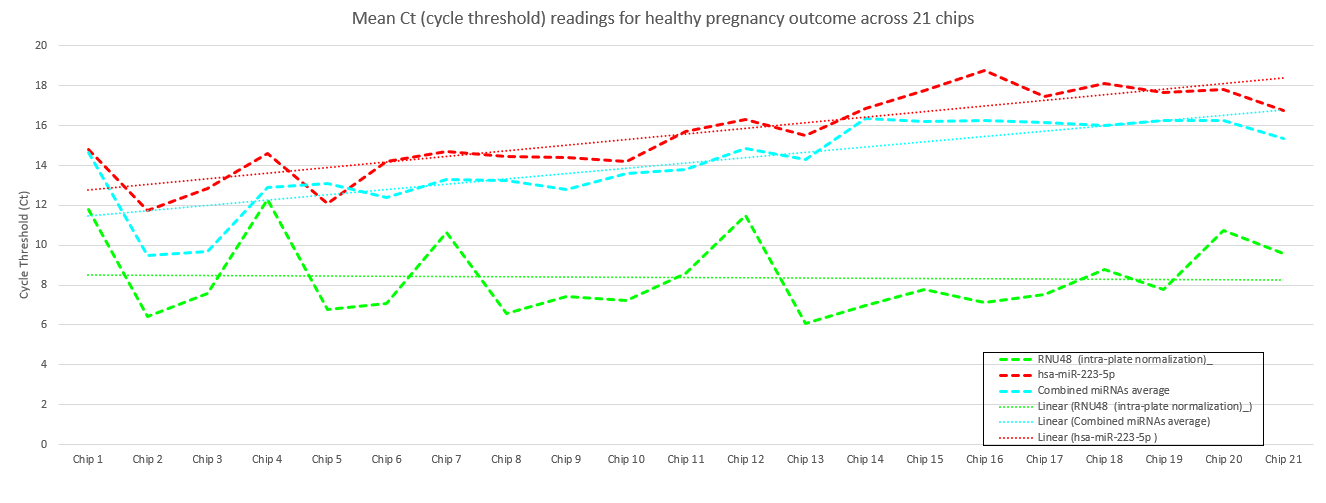


**S2 Figure:** Mean rt qPCR Ct (cycle threshold) readings for healthy pregnancy outcome across 21 plates (dashed lines) are displayed with associated trendlines (dotted lines). Trendlines have been added to illustrate the linear correlation between miR-223-5p (red dashed line) and combined average microRNA readings (blue dashed line) seen across plates. This supports the value of miR223-5p as an inter-plate control for microRNA reading ‘Batch effect” correction. Though RNU48 was an effective intra-plate control, RNU48 did not follow the combined microRNA trendline across plates (green dashed line), so was not used as an inter-plate control.
