# Supplementary material for: MicroRNAs isolated from peripheral blood in the first trimester predict spontaneous preterm birth
Source: PLoS One. 2020 Aug 13;15(8):e0236805. doi: 10.1371/journal.pone.0236805 (PMC7425910; doi:10.1371/journal.pone.0236805)
Supplement: S3 Fig — (DOCX) [file pone.0236805.s006.docx]

**S3 Figure.** The 12- microRNA panel identified preterm birth risk in women with no prior history of preterm birth.

| **Gest. age range at sample collection** | **No prior prematurity**  **Preterm Risk Score**  **6.6-12.9 weeks** |
| --- | --- |
| Sample size | 67 |
| Positive group (Preterm birth) | 6 |
| Negative group (Healthy) | 61 |
| Area under the ROC curve (AUC) | 0.76 |
| Standard Error | 0.107 |
| 95% Confidence interval | 0.17 to 0.74 |
| Significance level P (Area=0.5) | 0.0001 |
| Sensitivity | 83 |
| Specificity | 69 |
